# Automated Nuclear Analysis of *Leishmania major* Telomeric Clusters Reveals Changes in Their Organization during the Parasite's Life Cycle

**DOI:** 10.1371/journal.pone.0002313

**Published:** 2008-06-11

**Authors:** Fernando de M. Dossin, Alexandre Dufour, Elodie Dusch, Jair L. Siqueira-Neto, Carolina B. Moraes, Gyong Seon Yang, Maria Isabel Cano, Auguste Genovesio, Lucio H. Freitas-Junior

**Affiliations:** 1 Systems Biology of Pathogens Group, Institut Pasteur Korea, Seoul, South Korea; 2 Image Mining Group, Institut Pasteur Korea, Seoul, South Korea; 3 Departamento de Microbiologia, Imunologia e Parasitologia, Universidade Federal de São Paulo, São Paulo, Brazil; 4 Departamento de Genética, Instituto de Biociências, Universidade Estadual Paulista, Botucatu, São Paulo, Brazil; University of Liverpool, United Kingdom

## Abstract

Parasite virulence genes are usually associated with telomeres. The clustering of the telomeres, together with their particular spatial distribution in the nucleus of human parasites such as *Plasmodium falciparum* and *Trypanosoma brucei*, has been suggested to play a role in facilitating ectopic recombination and in the emergence of new antigenic variants. *Leishmania* parasites, as well as other trypanosomes, have unusual gene expression characteristics, such as polycistronic and constitutive transcription of protein-coding genes. *Leishmania* subtelomeric regions are even more unique because unlike these regions in other trypanosomes they are devoid of virulence genes. Given these peculiarities of *Leishmania*, we sought to investigate how telomeres are organized in the nucleus of *Leishmania major* parasites at both the human and insect stages of their life cycle. We developed a new automated and precise method for identifying telomere position in the three-dimensional space of the nucleus, and we found that the telomeres are organized in clusters present in similar numbers in both the human and insect stages. While the number of clusters remained the same, their distribution differed between the two stages. The telomeric clusters were found more concentrated near the center of the nucleus in the human stage than in the insect stage suggesting reorganization during the parasite's differentiation process between the two hosts. These data provide the first 3D analysis of *Leishmania* telomere organization. The possible biological implications of these findings are discussed.

## Introduction

The study of nuclear organization is essential to understanding the way genomes function. Spatial localization of a gene within the nucleus can modulate its expression, leading either to its activation or repression [1]. Chromosomes were first shown to be organized and later shown to occupy particular territories in the nucleus; chromosome properties such as size and gene density were found to be important in the nuclear positioning of the chromosome. In fact, a correlation between transcriptional silencing and localization to the nuclear periphery has been suggested. Gene-rich chromosomes have been observed to occupy the interior of the nucleus, while gene-poor chromosomes have been seen to localize at the nuclear periphery (for review see [2] and [3]).

In yeast, the interaction between the chromosome and nuclear periphery can be mediated by telomeres [4]. Telomeres are DNA-protein complexes at the physical ends of the chromosomes that function to protect chromosomal extremities against end-to-end fusions and degradation by nucleases. They are also important for the replication of chromosomal ends.

Telomeres show a conserved structure of G-rich tandemly repeated DNA sequences extending toward the chromosome extremities and ending in a 3′ overhang. The telomere repeat sequence 5′-TTAGGG-3′ is shared between phylogenetically unrelated organisms, such as vertebrates, and early diverging eukaryotes, such as the trypanosomatids [5].

Trypanosomatids are flagellated protozoa of medical importance as the causes of parasitic diseases such as leishmaniasis, Chagas' disease, and African trypanosomiasis. Of these three diseases, leishmaniasis is the most geographically widespread: it is present in over 80 countries and puts around 350 million people worldwide at risk of infection (WHO/TDR). There are over 20 *Leishmania* species pathogenic to humans, and no vaccines exist against any of them. The available treatments frequently show low efficacy and considerable toxicity.

Trypanosomes have peculiar biological features such as polycistronic transcription and *trans*-splicing. In the *trans*-splicing reaction, long polycistronic messages are processed by addition of a 30–40 nucleotides RNA derived from the spliced leader gene at the 5′ end of each cistron, followed by addition of a poly(A) tail at the 3′ end. Transcription is constitutive for almost all genes characterized to date and overall transcription rates vary according to the parasite developmental stages [6]. Thus, most regulation of gene expression in trypanosomes seems to occur posttranscriptionally, either by modulation of the stability of the processed mRNAs or by translational control (reviewed in [7]).

The life cycle of the *Leishmania* parasite comprises two stages: amastigote, the intracellular stage found in mammalian cells (human stage); and promastigote, the extracellular stage found in the insect vector (insect stage). The most studied species, *Leishmania major*, has a 32-megabase genome and 8200 protein-coding genes distributed on 36 chromosomes [8].

Telomeres in *Leishmania* are known to be heterogeneous in structure [9] and unlike what is found in other pathogenic protozoa, *Leishmania major* subtelomeric regions do not contain genes coding for the surface molecules frequently associated with parasite virulence [10]. Instead, *L. major* contains clusters of housekeeping genes extending up to 5 kb away from the telomeres [8].

The telomeric localization of virulence genes could provide increased opportunities to generate variability, as it is suspected of enhancing recombination creating new antigenic variants in *Trypanosoma brucei* and *Plasmodium* [11], [12], [13], [14]. In this process, the nuclear architecture may play a role in increasing the emergence of new antigenic and adhesive variants, in the same way that has been suggested for *P. falciparum*. The telomeres of *P. falciparum* lie in clusters of 4–7 chromosome ends in the nuclear periphery, and this clustering is thought to enhance recombination of subtelomeric genes like those of the *var* gene family [14].

Little is known about nuclear organization in *Leishmania* parasites. Given that these parasites are devoid of antigenic variation and their subtelomeric regions do not harbor virulence genes as seen for other protozoan parasites, we wanted to know whether *Leishmania* telomeres are organized in clusters. In addition, given that transcription in these parasites is polycistronic and constitutive, we wanted to know the distribution of the *Leishmania* telomeres in the nucleus, since in other models telomeres are often seen at the nuclear periphery associated with transcriptional silencing. To answer these questions we investigated the spatial organization of *Leishmania major* telomeres in the insect stage, and we extended this analysis to the intracellular human stage. The small-sized nucleus and complex telomere hybridization patterns in this organism made it impossible to study telomere dynamics using available methods. In order to have more accurate measures and obtain robust statistics on telomere localization within the nucleus, we developed a fully automated 3D image processing system to extract nuclei and detect telomere.

In this paper we describe the telomere organization found in *Leishmania* parasites, we compare the organization/distribution found in nuclei in the human stage and the insect stage, and we discuss the possible implications of these findings for understanding the biology of the parasite.

## Materials and Methods

### Parasites

*Leishmania major* MHOM/IL/81/Friedlin promastigotes (insect stage) were cultivated in M199 (Sigma) with 40 µM HEPES (pH 7.5), 20 µg/ml gentamicin, and 10% heat-inactivated fetal bovine serum at 28 °C.

### Amastigote preparation (human stage)

Mouse macrophage cell line J774A.1 was maintained in RPMI 1640 with l-glutamine (300 mg/L), 25 mM HEPES (pH 7.5) (GIBCO), 100 units/ml penicillin, 100 µg/ml streptomycin, and 10% heat-inactivated fetal bovine serum at 37 °C and 5% CO_2_. Macrophages were infected with exponentially growing promastigotes diluted to 2×10^5^ cells/ml and cultured 5 days prior to infection to allow them to reach stationary phase. The cells were then harvested and washed with RPMI 1640 medium before infection. In a 24-well plate, macrophages were added at 1×10^5^ cells/ml and incubated for one day prior to infection. On the day of infection, cells were washed once with medium and infected at a ratio of 1∶10 (host cell∶parasite) for 48 h at 37 °C and 5% CO_2_. After this period, cells were washed 3 times with PBS and processed for telomere detection as follows.

### Fluorescence *in situ* hybridization (FISH)

*Leishmania major* telomeres were detected by FISH using a telomere PNA probe (Telomere PNA FISH kit/FITC, DakoCytomation) according to the manufacturer's protocol except for the fixation step, which was performed with 3.7% formaldehyde (Sigma) for 15 minutes. In addition, the manufacturer's pre-treatment step was omitted.

To dissociate telomeric clusters, cells were treated with 0.05% proteinase K for 10 s prior to fixation [15], then submitted to FISH with the telomere PNA probe. Z-series images covering the whole nucleus were taken at distance intervals of 0.1 µm by exceeding the DAPI signal in a Nikon Eclipse 90i microscope using a 100×/1.4 Plan ApoVC lens and a Nikon DS-QiMc camera or a Zeiss LSM5 Line Scanning Confocal Microscope.

### Automated Image analysis

#### *A.* Nuclei detection and segmentation

First, all the parasites were automatically cropped from the 3D images by isolating each nucleus-kinetoplast pair. Then, a novel 3D analysis framework based on deformable models called “active meshes” was employed [16]. For each parasite, a mesh was used to detect the boundary of the kinetoplast, while another mesh was simultaneously used to detect the boundary of the nucleus. The mesh representation allowed the measurement of distances relative to the nuclear membrane and a 3D visualization that was fast and accurate.

#### *B.* Telomere cluster localization

Telomere clusters are small compared to the resolution limit of current optical microscopes. Therefore they appear on the images as the representation of the microscope's point spread function (PSF) [17]. This phenomenon is taken into account during the detection process, which consists of two steps. First, the image voxels with high curvature values are extracted. Then, a Gaussian approximation of the PSF is fitted to each pre-localized cluster to refine its localization in real space coordinates [18]. The intensity of each cluster is given by the estimated intensity value at cluster localization. For a proper comparison of the data from populations in the human or insect stages, the measured cluster intensities of each population were standardized by subtracting their respective population mean value, and dividing by their respective population standard deviation.

To compare the relationship between intensity and cluster position, a relative intensity value was calculated for each cluster. This relative intensity was calculated by dividing the cluster intensity value by the sum of all cluster intensity values from the same nucleus, thereby converting the intensity to a fraction of the whole cell intensity. This step made it possible to compare cluster intensities between different cells.

**Figure 1:**
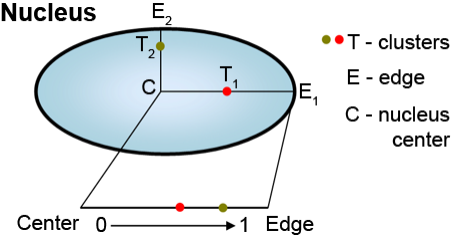
Scheme to illustrate determination of cluster position. Each cluster location T was projected on the detected membrane (mesh) using the vector formed by the center C of the nucleus and the cluster location T. This projected point E was then used to compute the ratio CT/CE, there normalizing all the relative cluster locations across all experiments to a ratio between 0 and 1.

To study the spatial organization of the clusters in each population and across all nuclei, we computed a relative location of each cluster along the nuclear radius as shown in Figure 1.

## Results

### *Telomeres are clustered in* Leishmania *parasites*

In FISH, hybridization of probe to target DNA is obtained through somewhat harsh fixation steps, high denaturation temperatures, and stringent washing conditions, sometimes leading to disruption of nuclear ultrastructure [19] [20]. We hypothesized that by using lower hybridization temperatures, we could better conserve this ultrastructure. Compared to DNA probes, peptide nucleic acid (PNA) probes show improved hybridization characteristics: they hybridize efficiently at low ionic strength, and their hybridization is more specific and faster (30–45 min), allowing milder hybridization protocols and resulting in lower background. Furthermore, PNAs are resistant to both protease and nuclease degradation (reviewed by [21] and [22]).

**Figure 2:**
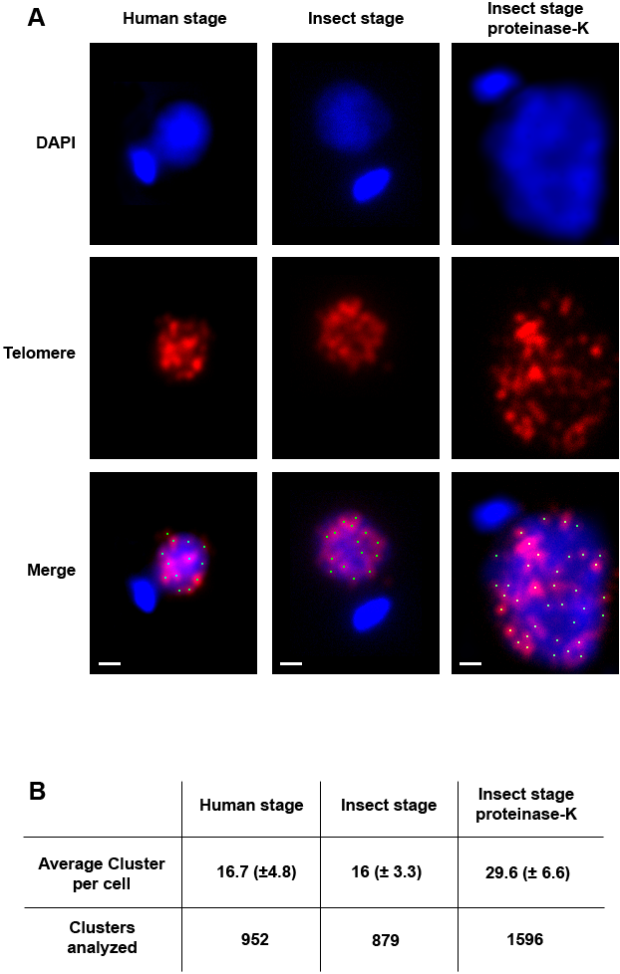
Telomeres are organized in clusters in *Leishmania major*. (A) Cells were analyzed by FISH using a fluorescent telomere PNA probe as described in Methods. DNA was stained with DAPI. 2D maximum intensity projection of Z-series showing telomeric clusters found in nuclei in the human stage (left column) and insect stage (middle column). Nuclei in the insect stage were treated with Proteinase K (right column) prior to fixation to examine whether telomeres are found in clusters. (B) Table showing the quantification of telomeric FISH foci as shown in (A). Bar, 1 µm.

Therefore, we decided to take advantage of PNA probes in order to study the 3D distribution of telomeres in *Leishmania* parasites. We performed FISH experiments in both *L. major* insect and human intracellular stages using a PNA probe complementary to the telomeric repeat of *Leishmania* parasites. As shown in Figure 2A, *L. major* telomeres were found in a speckled pattern, dispersed throughout the nucleus in both human and insect stages of the parasite.

Telomeres cluster with one another to form discrete foci tethered to the nuclear periphery, and this peripheral localization can modulate gene expression (reviewed by [23]). In order to verify whether *L. major* telomeres are organized in clusters, we performed proteinase K digestion of nuclei prior to cell fixation [15]. As expected, proteinase K treatment disrupted the overall nuclear structure. After this treatment, the number of telomere spots increased, indicating that telomeres were indeed associated in clusters, and that the clusters might have been disrupted by digestion.

The localization of telomeres was then assessed by automated image analysis of 3D series of *L. major* nuclei from insect and human stages. Determination of nuclear volume was carried out using the active mesh framework method (as described previously). One advantage of using this framework is that the Z-series are processed as a full volume, in contrast to methods in which each Z-slice is processed independently. Another advantage of our approach is that the meshes are permanently rendered during the detection process; thus there is no difference between what is seen on the screen and the model processing the data.

Interestingly, the observed average number of clusters in both stages is 16, suggesting that the number of clusters may be important for the parasite (Fig. 2B). Given that *L. major* has 36 chromosomes, an average of 4–5 chromosome ends are associated in each cluster. When we treated the cells with proteinase K before fixation, we obtained an average of 30 telomere spots per nuclei (Fig. 2B). These results provide evidence that *L. major* telomeres are in clusters brought together through protein interactions. Moreover, since *L. major* has 72 chromosome ends, our findings suggest that even after physical disruption of the clusters, telomeres may remain in close association. It is important to note that *Leishmania* chromosomes have never been observed in condensed states and thus the PNA-FISH system cannot be tested on *L. major* metaphase plates. Therefore, it is not possible to know precisely the resolution limit of this technique for detecting *L. major* telomeres. Nevertheless, it has been shown to detect more than 90% of telomeres in metaphase plates preparations from mammals [24], [25].

### Differences in telomere cluster location unveiled through precise automated assignment of cluster nuclear position

**Figure 3:**
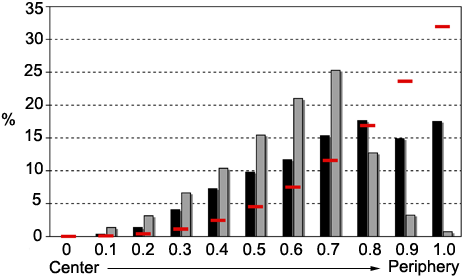
Telomeric clusters are widespread in the nucleus but concentrated in central areas. Comparison of the spatial distribution of telomeric clusters in nuclei in the human stage (gray bars, n = 952) and insect stage (black bars, n = 879) relative to the center of the nucleus (defined as position zero). The red lines show the values expected for random distribution. Below, the arrow illustrates the clusters distribution towards the nuclei.

**Figure 4:**
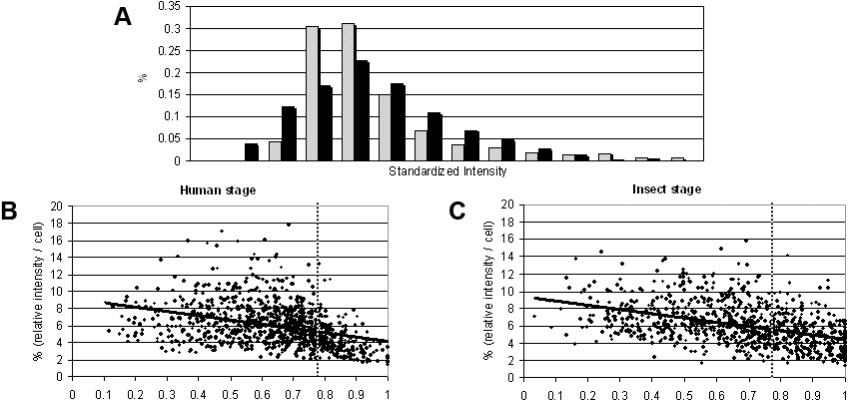
Telomeric clusters are reorganized between the human and insect stages. (A) Standardized intensity comparison of telomeric clusters between human stage (gray bars) and insect stage (black bars). (B and C) Correlation between the cluster intensity (intensity percentage relative to each cell) and relative cluster position in nuclei at the human stage (B) and insect stage (C). The values in the abscissa represent the relative distance of the clusters in relation to the center of the nucleus, defined as position zero. The trend lines are adjusted to linear regressions, and the dotted lines define the radius used to divide the nucleus in two parts of identical volume, one being internal and the other external.

After measuring the positions of hundreds of clusters in the nuclei of both *L. major* stages, we decided to analyze the distribution of the clusters. Nuclei from human and insect stages were identified based on DAPI signal and segmented as described in the Methods section. Each telomeric cluster was assigned a relative location along the nuclear radius. Even though telomere clusters are widespread throughout the nucleus during both stages, they concentrate in central areas more frequently than would be expected for random distribution, as shown in Figure 3. Moreover, the position of the telomere clusters differs between the two stages: clusters are more concentrated near the center of the nucleus in the human stage than in the insect stage. Another way of looking at these data is to divide the nucleus in two parts of identical volume, one being internal and the other external (Figures 4B and 4C, dotted vertical lines). This procedure reveals that ∼85% of clusters in the human stage, but ∼50% of clusters in the insect stage, are distributed in the internal half of the nuclear volume. Thus, transition to the human stage reduces the fraction of clusters in the external half of the nuclear volume to 15%, which means that the telomeres are repositioned to the center of the nucleus.

This suggests a spatial reorganization of *L. major* telomere clusters upon transition between stages of the parasite life cycle.

### Analysis of telomeric cluster intensity suggests reorganization between human and insect stages

The observed spatial reorganization of telomeric clusters between insect and human stages prompted us to investigate whether the composition of clusters also changed between the two stages.

The intensity of a telomeric cluster depends on the number of telomeric repeats present on each cluster. The number of repeats, however, can be attributed to the number of repeats within a single chromosome extremity as well as to the number of extremities present in each cluster. To our knowledge, there is no evidence so far that the repeat number within the chromosomes changes between insect and human stages. We therefore assume that the intensity of telomeric clusters depends solely on the number of chromosomes associated in each telomeric cluster.

For proper comparison of the data from human and insect stages, the measured cluster intensities of each population were standardized by subtracting their respective population mean value, then dividing by their respective population standard deviation. Figure 4A shows a comparison of the standardized cluster intensity distribution between human and insect stages. Surprisingly, the overall pattern of clusters intensity changes from one stage to the other, suggesting that chromosomal distribution in telomeric clusters changes upon *L. major* cellular differentiation.

To further gain insights into telomere distribution among the clusters, we correlated the intensity of clusters to their nuclear position. In both insect and human stages, the most intense clusters tend to be centrally located (Figures 4B and 4C). At present it is not known whether this differential distribution is biologically relevant, and whether it is due to differences in the distribution of chromosomes according to telomere size, or to a different number of chromosomes per telomeric cluster.

**Figure 5:**
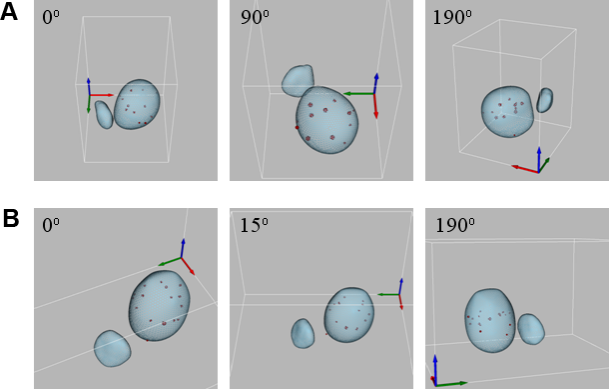
Three-dimensional views of an *L. major* nucleus showing the spatial distribution of telomeric clusters. Nucleus and kinetoplast are shown in blue and telomeric clusters in red. Differences in cluster intensity reflect differences in cluster size. The views are rotated clockwise with the angles indicated at the top of each panel. (A) Human stage nucleus. (B) Insect stage nucleus. Bar, 1 µm.

In order to facilitate the visualization of spatial distribution of telomeres in *Leishmania* parasites, 3D models were produced for both the human and insect stages (Figure 5A and 5B, respectively, and supplemental movies S1 and S2).

## Discussion

Here we have shown that *Leishmania major* telomeres are organized in clusters in both stages of the parasite life cycle. We have also observed that the number of clusters per cell does not change between the different life stages, suggesting that it may be important for parasite nuclear biology. Interestingly, the positioning of these clusters in the nucleus changes from one stage of the life cycle to the other. In the human stage, the clusters are more concentrated in the nucleus center, although in both stages clusters are found throughout the whole nuclear space. A comparison of cluster intensities shows that there is a reorganization of the nucleus when the parasites differentiate from one stage to the other, with cluster intensities being more homogeneous in the human stage. In both stages, however, we show that the clusters with the highest intensities are kept at more internal positions in the nucleus, while clusters of lower intensities localize towards the periphery.

The organization of *Leishmania* telomeres in clusters is comparable to the situation observed in other eukaryotic cells. In yeast, telomeres are clustered and tethered to the nuclear periphery. Association with the nuclear periphery correlates with gene silencing at the telomeres, where genes are closer to the pools of silencing proteins such as the Sir proteins. This transcriptional inhibition due to the telomeric localization of a gene is called the telomere position effect (TPE), and association of the telomeres with the periphery is thought to be necessary for TPE to occur (reviewed in [26]). For example, telomeric repression in trypanosomes has been demonstrated for genes encoding variant surface molecules (VSG) in *T. brucei* [27]. However, the importance of the nuclear localization in this process remains unclear. It has been suggested that perinuclear localization facilitates transcriptional repression in the stage of the parasite that does not express the VSG genes and that in order to be expressed, the VSG gene moves away from the periphery towards the center of the nucleus ([28] and for review see [29]).

We have observed that *Leishmania major* telomere clusters are not concentrated at the nuclear periphery but instead are distributed throughout the nucleus. Unlike what is seen for *Trypanosoma brucei*, *L. major* subtelomeric regions do not contain genes coding for surface molecules [8] [10]. The presence of housekeeping genes at the subtelomeric regions of *Leishmania* may explain the distribution of the telomeres throughout the nucleus.

Besides the lack of perinuclear localization of the telomeres, we have also observed that *Leishmania major* telomeres are reorganized in the nucleus during the life cycle. In *T. cruzi*, an extensive redistribution of the heterochromatic regions occurs during the life cycle and is associated with changes in the transcriptional status of the cell [6]. We have shown that in both stages of the parasite life cycle the more intense clusters are found in central positions and clusters of lower intensity localize towards the periphery. The role of chromatin in this organization was not examined and therefore cannot be ruled out. It is possible that the decrease in cluster intensity reflects a difference in probe accessibility due to more compact heterochromatin in the periphery compared to more relaxed and accessible euchromatin in central regions. Whether the reorganization of *Leishmania* telomeres reflects a more extensive and general reorganization of the chromatin remains to be elucidated, as does the functional importance of the telomere reorganization itself.

## Supporting Information

Movie S1 Three-dimensional reconstruction of an *L. major* nucleus showing the spatial distribution of telomeric clusters in *L. major* human stage. Nucleus and kinetoplast are shown in blue and telomeric clusters in red. Differences in cluster intensity reflect differences in cluster size. This reconstruction was based in 3D images from a single parasite.
(2.15 MB MOV)

Movie S2 Three-dimensional reconstruction of an *L. major* nucleus showing the spatial distribution of telomeric clusters in *L. major* insect stage. Nucleus and kinetoplast are shown in blue and telomeric clusters in red. Differences in cluster intensity reflect differences in cluster size. This reconstruction was based in 3D images from a single parasite.
(2.72 MB MOV)
